# Does watching TV or videos during the day affect nighttime urination?

**Published:** 2024-03

**FEBRUARY 21, 2024 -** In a study published in Neurourology and Urodynamics, adults who spent 5 or more hours a day watching TV and/or videos were more likely to develop nocturia, or the need to urinate multiple times during the night.

The study drew from 2011–2016 data from the National Health and Nutrition Examination Survey. Among 13,294 US individuals aged 20 and older, 4,236 (31.86%) reported experiencing nocturia, while 9,058 (68.14%) did not. Participants with 5 or more hours of TV and/or video viewing time per day had a 48% higher risk of experiencing nocturia compared with those with less than 1 hour of daily TV and/or video viewing time.

“As individuals increasingly engage in screen‐based activities, a comprehensive understanding of the impact of extended TV and/or video time on patterns of nocturia is crucial for both healthcare professionals and public health practitioners,” the authors wrote. “For individuals who engage in prolonged TV and/or video time, healthcare professionals can offer behavioral intervention recommendations, encouraging appropriate screen time management.”

***URL upon publication: https://onlinelibrary.wiley.com/doi/10.1002/nau.25406***

***Full citation:** “Association between TV and/or video time and nocturia in adults: An analysis of the National Health and Nutrition Examination Survey.” Junwei Wang, Aiwei Zhang, Miaoyong Ye, Cunming Zhang. Neurourology and Urodynamics; Published Online: 21 February 2024 (DOI: 10.1002/nau.25406).*

*Copyright © 2019 The Cochrane Collaboration. Published by John Wiley & Sons, Ltd., reproduced with permission.*

